# Let It “B”? The Role of Hepatitis B Universal Vaccination among Italian Problematic Drug Users

**DOI:** 10.3390/ijerph120403979

**Published:** 2015-04-13

**Authors:** Fabio Lugoboni, Raimondo Maria Pavarin, Chiara Resentera, Daniele Gambini

**Affiliations:** 1 Department of Internal Medicine, Addiction Unit, Verona University Hospital, Verona 37134, Italy; E-Mail: chiara.resentera@gmail.com; 2 Department of mental health and addictions, Epidemiological Observatory on Pathological Addictions, Bologna 40121, Italy; E-Mail: raimondo.pavarin@ausl.bologna.it; 3 Department of mental health and addictions, Addiction Clinic, Bologna 40121, Italy; E-Mail: daniele.gambini@ausl.bologna.it

**Keywords:** HBV, hepatitis, vaccine, universal vaccination, substance abuse, drug use, addiction

## Abstract

Hepatitis B virus (HBV) hepatitis is extremely common among problematic drug users (DUs). As of 2012, 47 of the 53 European countries had implemented a universal hepatitis B vaccination programme, a scenario that could radically change its spread. Even so, drug users are still one of the main groups at risk of being infected by HBV, exposing the fact that universal vaccination still has not managed to reach an optimal level of contagion protection. In order to evaluate the role of universal HBV vaccination in protecting against risk behaviour related to the use of illicit drugs, a group of 748 DUs, 511 male and 237 female, was tested for HBV markers, at their first access to public addiction clinics in the metropolitan area of Bologna, Italy. 487 were born after 1981, so they were eligible to have received HBV vaccination in adolescence or at birth; in these subjects antibodies against HBV core antigen had the significant prevalence of 6.2%. Universal HBV vaccination has shown evidence of protecting against infection in the general population. These results, amongst the first to evaluate actual protection in DUs vaccinated at birth or during adolescence, show that compulsory universal vaccination does not solve the problem of HBV transmission in the most at risk groups and that additional strategies must be studied and implemented to address this issue.

## 1. Introduction

Illicit drug use and consequent infections are among the most significant problems in the world [1,2]. Viral hepatitis is common among drug users (DUs), not only those who inject the drug [3,4,5,6,7], presenting relevant consequences in morbidity and mortality along with high social and economic cost [3,7,8,9,10,11]. In DUs deaths from liver disease have increased significantly [12,13,14]. 

Chronic carriers have a 25% risk of dying from the consequences of HBV infection, such as cirrhosis and liver cancer [1,2,15]. Furthermore, HBV is the virus with the highest potential for sexual transmission and therefore the one that most of all tends to spread among the general population [1,2,3,4,5,6,7,16]. Vaccination is the most effective measure to prevent HBV infections and related consequences [10,16,17]. 

In 1991, the World Health Organization (WHO) recommended the integration of the HBV vaccine into the national immunization programs in countries with a high HBV carrier prevalence and in all other counties by 1997 [1,2,10,17,18]. As of 2012, 47 of the 53 European countries had implemented a universal HBV vaccination program. A comprehensive strategy to eliminate HBV transmission includes measures to further increase the coverage rate, close the gap between recommendations and routine practices, screen and treat chronically infected individuals, prevent breakthrough infections and encourage the screening and vaccination of previously unvaccinated adults at risk for HBV infection [17,18,19]. However, the incidence of acute HBV remains significant among adults, who accounted for 95% of approximately 43,000 new HBV infections in 2007 in the US [19]. The highest incidence of HBV acute infection has been found among adults aged 25–45, with 80% being associated with high-risk sexual behaviour or illicit drug-use. Injection-drug use accounts for approximately 16% of new HBV infections in the US. Risk of HBV infection among unvaccinated DUs is the highest and increases with the number of years of drug use [7,10,16,17,18,19,20]. 

The low percentage of vaccinated DUs substantially reflects two factors: the absence of specific vaccination programs and the small number of services dedicated to DUs capable of administering vaccinations [16,17,21,22,23,24,25,26,27,28]. In Italy, an HBV vaccination campaign for high-risk groups started in 1983. Despite the decreased circulation of HBV in the late 1980s, a compulsory universal vaccination against HBV was introduced for all newborns and for 12-year-old children (a double cohort policy of mandatory immunisation) in 1991, moving to compulsory vaccination of newborns only in 2003 [17,18,29,30]. In Italy, the prevalence of chronic HBV carriers decreased from 3% in 1980 to 0.9% in 1997 [17,29,30,31]. 

Substantial evidence suggests that after primary immunization with HBV vaccine, Anti-HBcAb levels decline slowly and 17%–50% of young adults vaccinated have low or undetectable concentrations of antibodies 10–15 years after vaccination, but even when protective antibody titre declines to <10 mIU/mL, nearly all vaccinated subjects are considered to remain protected against infection, probably through selective expansion of clones of antigen-specific B and T lymphocytes [17,29,30,31,32]. For these reasons most vaccinees retain immune memory and a lasting protection against HBV for up to two decades after completion of immunisation programs [17,29,30,33,34]. Evidence suggests that the immunological response in DUs is impaired compared to the general population. Dysfunction of cell-mediated immunity, alcohol use, polydrug abuse, smoking status, multiple bacterial infections, HCV/HIV positivity and malnutrition are all possible explanations for the lower immune response to HBV vaccination in DUs [7,10,16,28,35,36].

There is little data in the literature regarding antibody titre in young DUs that have been vaccinated during adolescence for HBV and, more importantly, regarding the actual capability of compulsory vaccination to prevent transmission between DUs.

Seroconversion is normally not verified after HBV vaccination because of the extremely high probability of this happening at a young age [1,2,17]. The need for booster doses in HBV vaccine programmes remains controversial. The duration of vaccine-induced immunity is uncertain, but it is supposed to be long-term (around 15–20 years) [37,38,39,40]. Several studies have reported that booster doses of childhood immunisations should be considered in adolescence [32,41,42,43,44], however a much greater number of studies have demonstrated that booster doses are not needed in immunocompetent individuals that have received a complete series of HBV vaccines [40,45,46,47,48]. It should be noted that these studies have only considered the general population, usually young. However, at present, the WHO does not recommend the universal administration of booster doses [1,2,31].

## 2. Experimental Section 

### 2.1. Aims

In Italy current adults between the ages of 19 and 32 should have been compulsorily vaccinated against HBV, having been administered the three doses when they were 12 years old. Below the age of 21 they should instead have been given the neonatal vaccination. Coverage of HBV vaccination has been estimated to be of 97% in Northern Italy, but only 60% in the South, with intermediate percentages in Central Italy [29,30,31,32,33,49]. The aim of this study is to estimate vaccinal protection against HBV infection in young DUs, using Anti-HBcAb as a marker of prior HBV infection.

A positive HBcAb test is compatible with several scenarios, depending on other markers: acute infection, recovery, chronic infection, false positive. The presence of HBcAb, even without considering the presence of other markers, is considered a valid indicator of prior HBV infection, in epidemiological studies [10,16,20,21]. We decided to study a group of DUs born in Northern Italy, an area with high coverage of HBV universal vaccination. The aims of our study were the following:

- Test for HBV prevalence in DUs screened at the Public Addiction Centres Centers (PACs) of Emilia region.
- Test for HBV prevalence among younger DUs, compatible with having received the mandatory vaccination against HBV.
- Compare the prevalence for HBV among different areas of origin of DUs, based on the probability of having or not having received universal vaccination.
- Test the prevalence for human immunodeficiency virus (HIV) and hepatitis C virus (HCV).

### 2.2. Methods

In this cross-sectional observational study subjects that for the first time turned to a Public Addiction Clinics (PACs) were selected and studied. 

Data sources—At the PACSs, 10 health services out of 27 operating in the Emilia region, a digital regional folder is used to collect data (personal data, serological data) at first admission. Collected data was sent to the Epidemiological Observatory on Pathological Addictions (EOPAs). Established in 2008, the mission of EOPAs is to provide objective, reliable and comparable information on drugs and drug addiction and their consequences, giving information to social and health services and local authorities (Regional Council Resolution 19 May 2008 n. 698).

EOPAs developed in the metropolitan area of Bologna, the capital of the region, and is expanding towards the towns. Since the purpose of data collection is the analysis of the results by the EOPAs, every person who undergoes the screening tests gives consent to the use of his anonymous data.

In Italy, with a population of 58,000,000, there are 569 PACs, each with a catchment area of some 100,000 inhabitants on average. Extremely different numbers of drug users attend the different centres, ranging from less than 100 to more than 1000 patients. These publicly funded PACs provide counselling, treatment for drug withdrawal, methadone and buprenorphine maintenance programmes, naltrexone programmes, psychotherapy and other services. The centres also provide care for medical problems related to addiction, HIV, HCV and HBV testing. 

### 2.3. Analyzed Variables

The following variables were used: age, sex, citizenship (Italian or foreign), birthplace of Italians (North, Center, South), date of birth (before or after 1981), HBV serology (HBsAg, Anti-HBsAb, Anti-HBcAb), HCV serology (antibodies), HIV serology (antibodies). 

Given the impossibility of retrieving participants’ vaccinal records, particularly those of subjects who were Anti-HBcAb positive, particular importance was given to the birthplace because of the unreliability of asking DUs directly whether they had been vaccinated [50,51,52], therefore it was chosen to assess a group of DUs born in Northern Italy, an area with a very high coverage of HBV vaccination. Citizenship, for the same reason, was evaluated because of the risk of uncertainty regarding vaccination and to exclude the possibility of childhood infection in people coming from endemic areas. HBV serology was assessed and divided into three groups as follows:

1 Positive HBsAg marker and/or positive Anti-HBcAb: test considered positive for current or prior HBV infection
2 Presence of isolated Anti-HBs Ab: test positive for vaccination
3 No positive marker: negative test

### 2.4. Statistical Analysis

A multivariate analysis using logistic regression was used and odds ratios and respective 95% C.I. were done to analyze the profile of the seroconverted subjects. Stata 11.0 was used for statistical analysis (StataCorp LP Statistical Software, College Station, TX, USA, 2009).

## 3. Results

Among the subjects that accessed PACs in 2012 (2588 DUs), 1679 DUs were excluded from the study because their HBV serology had not been tested, and another 161 that had been tested but the results had not been registered. The test had been performed in 35.1% of the cases. The studied group comprised of 748 subjects, 28.9% of the total. 261 subjects (34.9%) were born before 1981, 487 (65.1%) after 1981. The mean age, at their first PAC access, was 22.5 (SD 2.6), equally distributed in both sexes. 511 subjects were male (68.3%) and 237 were female (31.7%). Ninety five subjects were foreigners (mean age 22.8), making up 12.7% of the group, and 653 were Italian (mean age 22.4).

Among Italian subjects, 421 (64.6%) were born in the North of Italy (mean age 22.0), 59 (9.1%) in the Center (mean age 23.3), 172 (26.4%) in the South (mean age 23.3).

In the analyzed group, active HBV at the moment of testing (HBsAg presence) was present in 2% (15/748 DUs). The prevalence of contact with HBV (HBcAb positive) was instead 8.6% (64/748 DUs). In the 261 subjects born before 1981 this prevalence was 13% (34/261 DUs), while in the 487 subjects born after 1981 it was 6.2% (30/487 DUs) (*p* = 0.001) (Table 1). The prevalence of positive HIV serology was 1.3% (10/748 DUs) and positive HCV serology was 19.4% (145/748 DUs).

In the group of 653 Italian DUs the prevalence of HBV infection was 6.7% (44/653 DUs). In those born after 1981, Anti-HBcAb was found in 18/421 (4.3%) subdivided geographically as follows: North13/136 (9.6%), Center 1/18 (5.6%), South 12/78 (15.4%). In the Italian DUs born after 1981, therefore eligible to have received compulsory vaccination, the presence of protective antibodies (Anti-HBsAb), showing that they had been vaccinated, was detectable in 72.1% of the subjects susceptible to have come in contact with HBV (290/402 DUs).

**Table 1:** Anti-HBcAb seroconversion: univariate analysis.

Demographic Characteristics		Anti-HBcAb +		Odds Ratio	CI 95%	*p*
		No	Yes			
Total		684 (91.4)	64 (8.6)			
Sex	Females	213 (89.9)	24 (10.1)	1		0.296
	Males	471 (92.2)	40 (7.8)	1.33	0.78–2.26	
Citizenship	Italians	609 (93.3)	44 (6.7)	1		<0.0001
	Foreigners	75 (78.9)	20 (21.1)	3.69	2.05–6.65	
Birthplace (Italians)	North	401 (95.3)	20 (4.7)	1		0.023
	Center	53 (89.8)	6 (10.2)	2.27	0.87–5.91	
	South	154 (89.5)	18 (10.5)	2.34	1.21–4.55	
Year of Birth (Italians)	≥1981	403 (95.7)	18 (4.3)	1		0.001
	<1981	206 (88.8)	26 (11.2)	2.83	1.51–5.27	

### 3.1. Univariate Analysis

The univariate analysis showed that, foreigners had significantly higher prevalence of HBV infection compared to Italians. Considering Italians only, the probability of being infected was higher for those born in the South compared to those born in the North, and for those born before 1981.

### 3.2. Multivariate Analysis

A multivariate analysis using logistic regression was performed to outline the profile of the Italian seroconverted. The variables used were sex, year of birth and place of birth (North, Center, South) (Table 2).

**Table 2:** Anti-HBcAb seroconversion among Italians: multivariate analysis—logistic regression.

Demographic Characteristics		Odds Ratio	CI 95%	*p*
Sex	Females	1		
	Males	1.51	0.79–2.90	0.215
Year of Birth	≥1981	1		
	<1981	2.80	1.48–5.31	0.002
Zone of Birth	North	1		
	Center	2.26	0.86–5.96	0.099
	South	2.13	1.09–4.19	0.028

## 4. Discussion

This study found that the prevalence of Anti-HBc positivity among the Italian DUs was shown to be much lower compared to similar studies conducted in the past decade, confirming the expected decline of HBV even in these subjects, due to the universal immunization programs. The self-evident differences in contact with HBV, shown in this study, between DUs born in Northern Italy, where neonatal vaccination compliance is nearly total (97%, higher than the 88% percent the U.S.A. and 84% in Belgium) [53,54,55,56,57,58,59,60], and those born in the Center-South, with clearly lower vaccination coverage, could reflect an effective role played by universal vaccination. 

Secondly, this study seems to confirm what was recently observed among DUs in Australia, where despite a universal vaccination program that covered 90% of the non-adult population, a significant number of DUs (17%, theoretically already vaccinated during childhood) resulted exposed to contact with HBV [61]. Furthermore, the presence of protected antibodies in our group was significantly lower when compared to that found among students of Padua University Medical School (Northern Italy) [62]. 

Despite the availability of safe and effective HBV vaccines for more than 30 years, the burden of HBV is still substantial and vaccination delay has been described in several countries with unexpected regional differences. In the 2000−2002 in the US, the three-dose HBV vaccine among children ranged from 49.4% (Vermont) to 81.6% (Rhode Island) [53]. Paradoxically, universal vaccination has led the population of developed countries to feel protected and to believe that HBV is no longer a public health issue [31,54]. 

Thirdly, the finding that only a third of the DUs in our study had been tested for HBV markers is worrying. Testing has multiple functions: not only to monitor the phenomena, but also to develop attention in healthcare workers, in DUs and their families towards the infective risks linked to the use of illicit drugs [16,28]. The decline in new HIV infections and a drop on the use of the injective route in new DUs, paired with the idea that mass HBV vaccination has finally solved the problem in all the young population, may have led to this decline in testing, particularly for HBV. In 2011, in Italy, of the DUs that accessed PACs, only 30.5% was tested for HIV, 16.6% for HCV and 21.1% for HBV, percentages even lower that those found in this study [63]. It is a wanton “lowering of the guard”, since HBV is up to 100 times more infectious than HIV and is highly sexually transmissible. Indeed, even though the DUs in this study were relatively young, the high prevalence of Anti-HCV positive tests and though less so for HIV, show that contact with these viruses starts from the first years of addiction.In our study a consistent percentage of the group was made up by foreigners, most of which were from nations at high HBV risk [64,65,66]. This can be a factor of augmented viral circulation, as it has been reported for hepatitis A virus (HAV) in low endemic countries [67,68,69]. 

There is an underlying problem, when we face the problem of vaccinations addressed to DUs. One of the underlying problems of addressing vaccination issues for DUs is the lack of specific studies focusing on this group. Vaccination schedules are extrapolated from the general population and then applied to DUs. In the 30 years since the introduction of HBV vaccination, only a single cohort study has evaluated the clinical (not only immunological) effectiveness of the HBV vaccination among DUs, a much more relevant finding than any immunological evaluation [7,16,70]. 

The debate on the appropriateness of a possible booster in young adults, not considered necessary for the general population, would probably greatly benefit DUs, especially if young, previously vaccinated and who have never come into contact with HBV. The evidence that a simple dose of vaccine, in those that received the universal vaccination, can substantially augment the antibody titre, even when this had disappeared, is encouraging [7,16,28]. Administration of a single dose is much less cumbersome for PACs compared to the traditional 0, 1, 6 months schedule, in a similar way to what has been shown to be effective for rapid vaccination in DUs during HAV outbreaks [69,71,72]. A potentially effective scheme against HBV in young DUs, that should be applied by PACs, could be implement a routine policy of “don’t ask, draw a blood sample, vaccinate and try to schedule another visit” [16,28,73,74], since DUs’ self-reporting is unreliable. Rapid vaccination protocols, specifically developed for DUs, have always shown better better uptake and completion rates than traditional protocols, established for the general population [16,28,75,76,77]. Additional HBV vaccine doses have always been shown to be safe, also in DUs [7,10,16,28]. Another strategy, more complex and expensive but with undoubted advantages for DUs with chronic hepatopathy, HCV-correlated or of other origin, could be to administer a combined HAV and HBV vaccine. Since 1996, in addition to the normal monovalent vaccines, a combined vaccine against both viruses, with a proposed schedule at 0, 1, 6 months, has been available (Twinrix^®^), which has been shown to be safe and immunogenic in the general population [78,79]. The combination of hepatitis A and B antigens in one single vaccine could offer the following advantages: fewer injections for protection against two infections, better compliance, lower implementation costs, and fewer missed vaccination opportunities [28,80,81,82]. 

However, this study has certain limitations. The diagnostic methods used for HBV, HCV and HIV cannot be specified because such information is unavailable, given the study’s design: a multicentre observational study. Significant information such as the type of drug or the route of administration was not reported. Even though it has been established, in regard to HBV, that is limitative to divide DUs in injectors and non-injectors and on the basis of the drug used [83,84,85,86], this information can help to better define the early risk to which young DUs are exposed. 

## 5. Conclusions 

There is a precise and disturbing finding in reviewing the scientific literature on vaccination in DUs: this group is at highest risk for vaccine-preventable hepatitis but it commonly receives little attention at all levels, both in terms of clinical management and scientific speculation. For at least twenty years DUs have been reported to respond very differently to immunization for reasons not yet completely understood. One of the problems is the fact that many articles describe the difficulties linked to vaccination in DUs but very few provide concrete experiences, for example data regarding immune response to vaccines. In the most recent systematic review on HBV vaccine intervention studies reporting seroprotection rates in DUs, of 978 citations reviewed, only 11 studies were eligible and included for final analysis [10]. The lack of data on the impact that universal vaccination can have on DUs is part of this context of culpable neglect; further studies and innovative methods are needed to improve protection against hepatitis, among DUs.
